# Probiotics as *Vibrio*-suppressing agents in *Litopenaeus vannamei* culture: A comprehensive review

**DOI:** 10.1016/j.cirep.2026.200303

**Published:** 2026-07-25

**Authors:** Tashrif Mahmud Minhaz, Sayeed Hasan Al Banna Tanim, Samiya Sultana Lima, Shahadat Hossain, Zahidul Islam, Sadia Afrin, Md Azharul Hoque Shakil

**Affiliations:** a Department of Aquaculture, Chattogram Veterinary and Animal Sciences University, Khulshi, 4225, Chattogram, Bangladesh; b Department of Marine Bioresource Science, Chattogram Veterinary and Animal Sciences University, Khulshi, 4225, Chattogram, Bangladesh; c Marine Fisheries and Technology Station, Bangladesh Fisheries Research Institute, Cox’s Bazar Sadar, 4700, Cox’s Bazar, Bangladesh

**Keywords:** Shrimp, Vibriosis, Biological control, Bacteria, Immunity

## Abstract

The Pacific white shrimp, *Litopenaeus vannamei*, is the most widely farmed shrimp species globally, contributing significantly to seafood production and coastal economies. However, intensified shrimp farming has increased disease outbreaks caused by pathogenic *Vibrio* species, particularly *Vibrio parahaemolyticus, Vibrio harveyi*, and *Vibrio alginolyticus*. These bacteria are responsible for diseases such as Acute Hepatopancreatic Necrosis Disease, vibriosis, and other infections that cause high mortality and reduced growth. Although antibiotics have traditionally been used to control bacterial diseases, their excessive and indiscriminate application has led to antimicrobial resistance, environmental contamination, and food safety concerns. As a result, environmentally sustainable alternatives such as probiotics have gained attention for improving shrimp health and suppressing pathogens. Probiotic microorganisms, including specific strains of *Bacillus, Lactobacillus, Pseudoalteromonas spp****.*** (CDM8 & CDA22), and *Clostridium* (*C. butyricum* CBG01), help maintain microbial balance, inhibit pathogen growth, and stimulate host immunity. Their protective mechanisms include competitive exclusion, antimicrobial compound production, interference with bacterial quorum sensing, and modulation of innate immune responses. Probiotics also enhance digestion, improve water quality, stabilize gut microbiota, and promote shrimp growth and survival. The integration of probiotics with modern practices such as biofloc technology and zero-water-exchange systems further strengthens disease management and environmental sustainability. This review summarizes the role of probiotics as *Vibrio*-suppressing agents in *L. vannamei* culture and highlights emerging strategies for sustainable shrimp aquaculture.

## Introduction

Shrimp aquaculture has experienced rapid global expansion over the past three decades and has become one of the most valuable sectors of the aquaculture industry. Among cultured shrimp species, the Pacific white shrimp *Litopenaeus vannamei* has emerged as the dominant species due to its fast growth rate, high adaptability to diverse environmental conditions, and tolerance to varying salinity levels [1,2]. Originally native to the eastern Pacific coast of the Americas, *L. vannamei* has been widely introduced to many Asian and Latin American countries where it now accounts for the majority of farmed shrimp production [3,4]. The global success of this species is largely attributed to technological advancements in hatchery production, selective breeding programs, improved feed formulations, and the adoption of intensive farming systems [[5], [6], [7]].

Despite its economic importance, the rapid intensification of shrimp farming has resulted in significant disease challenges. Pathogenic microorganisms, particularly bacteria belonging to the genus *Vibrio*, are among the most serious threats to shrimp aquaculture [8]. *Vibrio* species are naturally occurring marine bacteria that can become opportunistic pathogens under unfavorable environmental conditions such as poor water quality, high stocking density, and nutritional stress [9,10]. Several pathogenic *Vibrio* strains have been identified as causative agents of shrimp diseases, including *Vibrio harveyi, Vibrio alginolyticus*, and *Vibrio parahaemolyticus* [[11], [12], [13], [14], [15], [16]]. These bacteria can infect shrimp through the digestive tract or damaged exoskeleton, leading to systemic infections characterized by lethargy, tissue necrosis, reduced feeding, and high mortality rates [17]. One of the most devastating bacterial diseases affecting *L. vannamei* farming is acute hepatopancreatic necrosis disease (AHPND), also known as early mortality syndrome (EMS) [18,19]. This disease is caused by specific strains of *Vibrio parahaemolyticus* that harbor plasmid-encoded toxin genes responsible for severe hepatopancreatic damage in shrimp [[20], [21], [22]]. Since its first emergence in Southeast Asia in the early 2010s, AHPND has caused substantial economic losses to shrimp farmers worldwide [23]. Other forms of vibriosis also contribute significantly to production losses by impairing shrimp health and reducing survival rates [[24], [25], [26]].

Historically, antibiotics have been extensively used to control bacterial infections in shrimp aquaculture. Antimicrobial agents such as oxytetracycline, chloramphenicol, and sulfonamides have been applied either through medicated feed or directly into culture ponds [27]. Although antibiotics can temporarily reduce bacterial loads, their long-term effectiveness has become increasingly limited due to the rapid development of antibiotic-resistant bacterial strains [20]. Moreover, the overuse of antibiotics can lead to environmental pollution, disruption of beneficial microbial communities, and accumulation of drug residues in shrimp tissues, posing potential risks to human health [[28], [29], [30], [31]]. As a result, China, Thailand, Vietnam, Brazil, Chile, Bangladesh, Norway, Philippines and India have implemented strict regulations restricting the use of antibiotics in aquaculture production systems [[32], [33], [34], [35], [36], [37]]. In response to these challenges, researchers and aquaculture practitioners have explored alternative approaches for disease prevention and health management in shrimp culture. Among these approaches, probiotics have gained considerable attention as environmentally friendly and sustainable biological agents [38]. Probiotics are defined as live microorganisms that, when administered in adequate amounts, confer health benefits to the host organism. In aquaculture systems, probiotics can be introduced through feed supplementation, water application, or biofloc systems to improve microbial balance and enhance host immunity [[39], [40], [41]].

The beneficial effects of probiotics in shrimp aquaculture are mediated through multiple mechanisms. These microorganisms can compete with pathogenic bacteria for nutrients and ecological niches, thereby preventing pathogen colonization [42,43]. Many probiotic strains are also capable of producing antimicrobial compounds such as bacteriocins, organic acids, hydrogen peroxide, and lytic enzymes that inhibit the growth of pathogenic *Vibrio* species [44]. In addition, probiotics can interfere with quorum sensing systems that regulate virulence gene expression in pathogenic bacteria, thereby reducing their pathogenic potential [45,46]. Another important mechanism through which probiotics contribute to shrimp health is the stimulation of innate immune responses. Unlike vertebrates, shrimp lack an adaptive immune system and rely primarily on innate immune defenses to combat infections [47]. Probiotic supplementation has been shown to enhance several immune parameters in shrimp, including phenoloxidase activity, respiratory burst, lysozyme activity, and the production of antimicrobial peptides such as anti-lipopolysaccharide factors [48]. These immune enhancements improve the shrimp’s ability to resist bacterial infections and increase survival rates during disease outbreaks. Furthermore, probiotics play a crucial role in maintaining the stability and diversity of gut microbiota. The gastrointestinal tract of shrimp hosts a complex microbial community that influences digestion, nutrient absorption, and immune regulation [[49], [50], [51]]. Disruption of this microbial balance can favor the proliferation of opportunistic pathogens such as *Vibrio*. Probiotic supplementation can restore microbial equilibrium by promoting beneficial bacteria while suppressing harmful microorganisms [52,53,43]. This microbial modulation contributes to improved feed utilization, enhanced growth performance, and better disease resistance in cultured shrimp.

Recent developments in sustainable aquaculture practices have also emphasized the integration of probiotics with advanced culture technologies such as biofloc systems and zero-water-exchange farming [54]. These systems rely heavily on microbial processes to recycle nutrients and maintain water quality [55,56]. Probiotic bacteria introduced into such systems can enhance the stability of microbial communities, reduce the accumulation of toxic metabolites, and further inhibit pathogenic bacteria in the culture environment [[57], [58], [59]].

Although numerous studies have demonstrated the beneficial effects of probiotics in shrimp aquaculture, several knowledge gaps remain regarding their optimal application strategies, strain specificity, and long-term ecological impacts. Understanding the complex interactions between probiotics, pathogens, host immunity, and environmental factors is essential for developing effective disease management strategies in intensive shrimp farming systems [60]. This review aims to provide a comprehensive overview of the role of probiotics as *Vibrio* suppressing agents in *L. vannamei* culture. It discusses the current status of shrimp aquaculture, the challenges associated with bacterial diseases, and the mechanisms through which probiotics contribute to pathogen control and host health. Additionally, the review highlights emerging strategies and future perspectives for the sustainable intensification of shrimp aquaculture through probiotic-based interventions.

## Present status and opportunities of farming *Litopenaeus vannamei*

The Pacific white shrimp *L. vannamei* currently represents the most extensively cultured shrimp species in global aquaculture [[61], [62], [63]]. Since its introduction into Asian aquaculture systems in the late 20^th^ century, the species has rapidly replaced several native shrimp species due to its superior growth performance, high survival rates, and tolerance to a wide range of environmental conditions [64,65]. Global shrimp aquaculture production has increased substantially over the last two decades, and *L. vannamei* now contributes the majority of the world's farmed shrimp supply [66,67].

Major shrimp-producing countries include China, Ecuador, India, Vietnam, Indonesia, and Thailand, which collectively account for a significant portion of global production [[68], [69], [70]]. Technological innovations in hatchery management, broodstock domestication, and selective breeding programs have facilitated the large-scale cultivation of *L. vannamei* in both traditional and intensive production systems [71]. The development of specific pathogen-free (SPF) broodstock has also played a crucial role in improving shrimp health and enhancing production efficiency [66].

The adoption of intensive farming systems such as biofloc technology, recirculating aquaculture systems (RAS), and super-intensive tank-based production has further increased the productivity of *L. vannamei* farming [72,73]. These systems enable farmers to maintain higher stocking densities while improving water quality management and reducing environmental impacts [54,74]. In addition, advances in formulated feeds and nutritional strategies have contributed to improved feed conversion ratios and faster growth rates in farmed shrimp [39]. Despite these technological advancements, disease outbreaks remain one of the primary constraints affecting global shrimp production [75]. Pathogens including viruses, bacteria, and parasites continue to cause significant economic losses in many shrimp-producing regions [76]. Among bacterial pathogens, species belonging to the genus *Vibrio* have been recognized as major contributors to disease outbreaks in shrimp farms [4]. Consequently, sustainable disease management strategies have become a central focus in modern shrimp aquaculture research [77].

The cultivation of *L. vannamei* offers numerous opportunities for the continued growth and sustainability of the global aquaculture industry. One of the key advantages of this species is its high adaptability to diverse environmental conditions, including a wide range of salinity levels and water temperatures [78,79]. This adaptability allows shrimp farmers to culture *L. vannamei* in both coastal and inland aquaculture systems, thereby expanding the geographical scope of shrimp production [78]. Another major opportunity associated with *L. vannamei* culture lies in its rapid growth rate and efficient feed conversion. Compared with many other shrimp species, *L. vannamei* can reach market size within a relatively short production cycle, which enhances economic profitability for farmers [[70], [80]]. The species also exhibits strong tolerance to high stocking densities, making it suitable for intensive and super-intensive farming systems that maximize production per unit area [81].

Advances in genetic improvement programs have further strengthened the potential of *L. vannamei* aquaculture [82]. Selective breeding programs have produced strains with improved growth performance, enhanced disease resistance, and better environmental tolerance [83]. The availability of domesticated broodstock has also reduced the reliance on wild shrimp populations, thereby contributing to the conservation of natural ecosystems [84]. Different innovative aquaculture technologies such as biofloc technology, integrated multi-trophic aquaculture, and recirculating aquaculture systems enable more efficient nutrient recycling and reduced water exchange requirements [85]. These systems can enhance production efficiency while minimizing environmental impacts associated with shrimp farming. Furthermore, increasing global demand for seafood continues to drive the expansion of shrimp aquaculture [86]. Shrimp is widely regarded as a high-value seafood commodity with strong consumer demand in international markets [87]. As capture fisheries face increasing pressure and declining stocks, shrimp aquaculture is expected to play a crucial role in meeting future seafood demand. Consequently, the sustainable development of *L. vannamei* culture has become a key priority, necessitating the adoption of effective and environmentally sustainable disease-management strategies [2].

## Farming challenges of *L. vannamei* culture

**Fig. 1:**
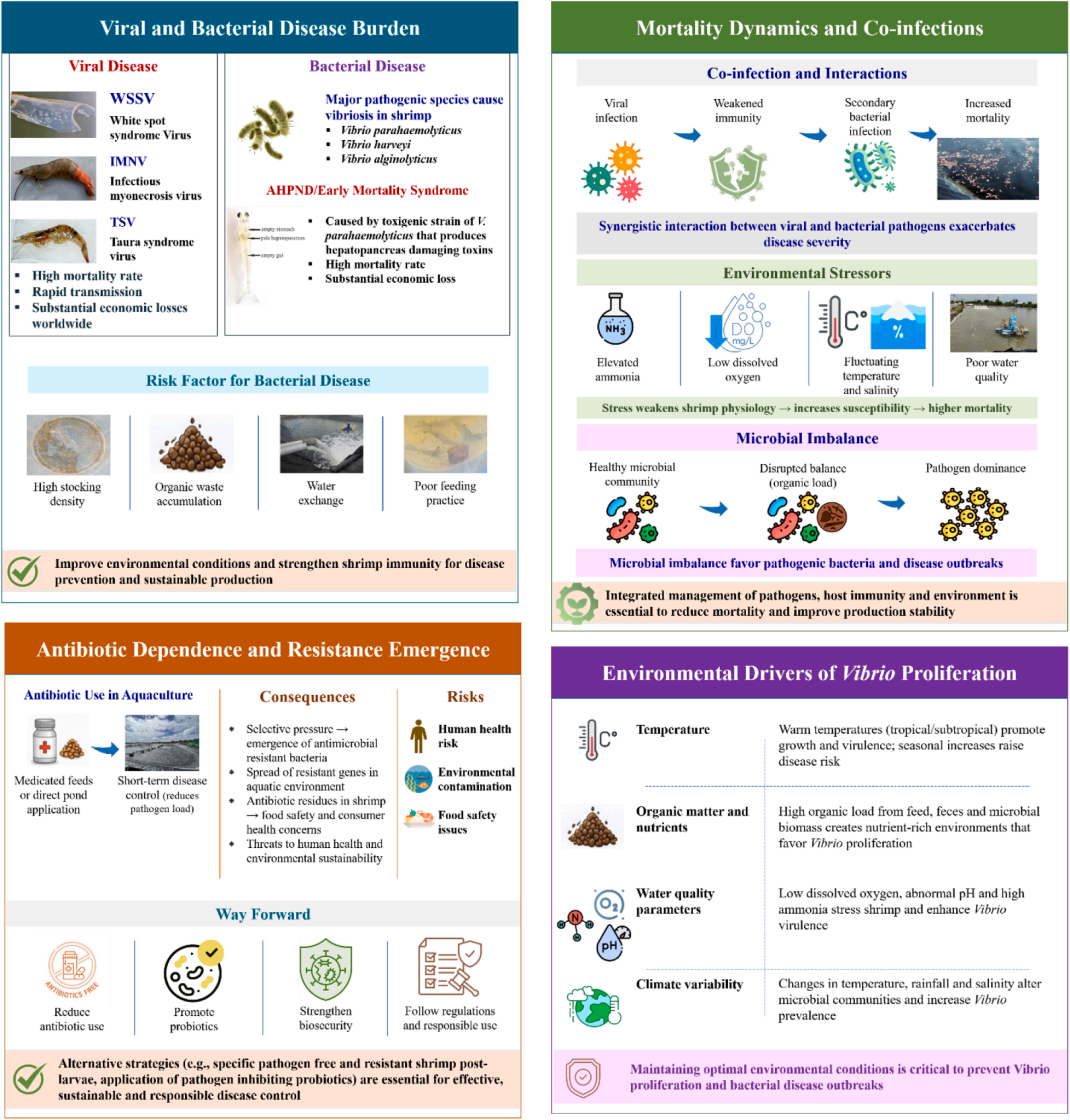
Diseases associated challenges of *Litopenaeus vannamei* farming.

Despite its considerable economic importance, the culture of *L. vannamei* faces numerous challenges that threaten the sustainability and profitability of shrimp farming operations (Fig. 1). One of the most significant challenges is the increasing prevalence of infectious diseases, which can cause large-scale production losses in shrimp farms worldwide [67]. Intensive culture practices, characterized by high stocking densities and increased organic loading, often create favorable conditions for pathogen proliferation [88].

Disease outbreaks represent one of the most significant constraints affecting the global shrimp aquaculture industry [75]. Both viral and bacterial pathogens have been responsible for substantial economic losses in shrimp farming systems over the past several decades [54]. Viral diseases such as white spot syndrome virus (WSSV), infectious myonecrosis virus (IMNV), and Taura syndrome virus (TSV) have historically caused large-scale mortality events in shrimp farms. In addition to viral pathogens, bacterial diseases caused primarily by *Vibrio* species have emerged as major threats to the health and productivity of cultured shrimp. Frequently reported co-infections involving bacterial, viral, and parasitic pathogens in intensive shrimp farming systems can exacerbate disease severity and lead to increased mortality rates [89]. Moreover, mortality dynamics is highly influenced by environmental stressors as well as imbalance of microbial communities [89,90]. The high prevalence of bacterial diseases in shrimp farms is often linked to environmental stress and poor farm management practices. Pathogenic *Vibrio* spp. bacteria are naturally present in marine and estuarine environments and can rapidly proliferate under favorable environmental conditions [91]. Factors such as high stocking densities, accumulation of organic waste, and inadequate water exchange can create favorable conditions for the proliferation of pathogenic bacteria [89,92]. On the other hand, antimicrobial agents are typically administered through medicated feeds or applied directly to culture ponds in an attempt to reduce pathogen loads [27]. Continuous exposure to antimicrobial agents creates selective pressure that favors the survival and proliferation of resistant bacterial strains [93,27]. Resistant bacteria can spread through aquatic ecosystems and potentially transfer resistance genes to other microbial populations [94,95]. Additionally, antibiotic residues may accumulate in shrimp tissues, raising concerns about food safety and consumer health [96]. Consequently, disease prevention strategies that focus on improving environmental conditions and strengthening shrimp immunity are essential for maintaining sustainable shrimp production [97].

Intensification of shrimp farming systems, on the other hand, are designed to maximize production through higher stocking densities, increased feed inputs, and greater management intervention [98]. While these systems can significantly increase shrimp yield per unit area, they also create conditions that may favor the rapid spread of infectious diseases [99,100]. Modern aquaculture systems such as biofloc technology (BFT) and recirculating aquaculture systems (RAS) have been developed to mitigate critical production risks by enhancing biosecurity, recycling nutrients, and stabilizing water quality [101,102]. Nevertheless, even advanced aquaculture systems are not completely immune to disease outbreaks. Effective disease management therefore requires a combination of improved biosecurity measures, microbial management strategies, and the use of beneficial microorganisms such as probiotics to maintain ecological balance within the culture environment [103].

## Probiotics in *L. vannamei* farming as *Vibrio* suppressors

The growing challenges associated with bacterial diseases and antibiotic resistance in shrimp aquaculture have intensified research into environmentally sustainable disease control strategies. Among these alternatives, probiotics have gained significant attention as biological agents capable of enhancing shrimp health and suppressing pathogenic microorganisms, particularly members of the genus *Vibrio* [97]. Probiotics are generally defined as beneficial microorganisms that, when administered in adequate quantities, confer health benefits to the host organism or improve the surrounding environment [104,43].

In shrimp aquaculture, probiotics can be applied through several routes, including incorporation into formulated feeds, direct addition to culture water, or integration within microbial management systems such as biofloc technology [105]. Common probiotic genera used in shrimp farming include *Bacillus, Lactobacillus, Pseudoalteromonas*, and *Clostridium* [[80], [106], [107], [108]] (Table 1). These microorganisms have demonstrated the capacity to improve shrimp growth performance, enhance immune responses, and inhibit the proliferation of pathogenic bacteria [[109], [110], [111], [112]]. One of the primary benefits of probiotic application in *L. vannamei* culture is the suppression of pathogenic *Vibrio* populations [113]. This inhibitory effect is achieved through multiple biological mechanisms including competition for nutrients and ecological niches, secretion of antimicrobial compounds, and interference with bacterial communication systems [114,115] (Table 1). By reducing pathogen abundance, probiotics contribute to lower disease incidence through boosted immunity, environmental stability and improved survival rates in shrimp farms. Certain probiotic bacteria possess extracellular hydrolytic enzymatic capabilities (protease, amylase, cellulase, lipase, and sometimes chitinase) that facilitate the degradation of organic matter and the transformation of nitrogenous waste products such as total ammonia nitrogen (TAN), nitrite-nitrogen (NO_2_^−^-N), and unionized ammonia (NH₃). These microbial activities can help maintain favorable environmental conditions and reduce stress on cultured shrimp [[116], [117], [118]].

**Table 1:** List of the most potential probiotic bacteria for *Vibrio* inhibition in *Litopenaeus vannamei.*

Bacteria/ Probiotic strain	Effective Concentration	Life stages of shrimp	Cellular or Molecular Method of *Vibrio* inhibition	Efficiency	References
*Bacillus subtilis* (K3)	1 × 10⁵ CFU.ml⁻¹ (in water)	Juvenile	Quorum quenching (via lactonase production), competitive exclusion, and modulation of intestinal microbiota. Reduced expression of *Vibrio* virulence genes. Increased immune parameters (THC, LYZ, SOD, CAT) and bactericidal activity. Up-regulated immune gene expression (proPO, LYZ, PEN).	80-95% survival across different *V. parahaemolyticus* strains challenge vs. 32.78% in control.	[119]
*Bacillus subtilis* (DSM 32315, Gutcare®)	5% inclusion in diet (equivalent to ∼2.5 × 10⁷ CFU/g feed)	Juvenile	Enhanced antioxidant capacity (SOD, CAT, T-AOC), increased immune enzyme activity (PO, LZM, ACP, AKP), and improved intestinal health.	Significantly reduced the abundance of *Vibrio* in shrimp gut.	[120]
*Bacillus subtilis* (IS02)	1 × 10⁸ CFU.ml⁻¹ (in water)	Larvae, post-larvae	Enhanced immune parameters (proPO, PE gene expression), digestive enzymes, and stress tolerance.	35.7% survival after 4 weeks vs. 27.3% in control; higher resistance to nitrite and ammonia stress.	[121]
*Bacillus subtilis* (BSXE-1601)	3.5 × 10^8^ CFU.g^−1^ feed	Juvenile	Production of antimicrobial peptides (bacillibactin, fengycin, surfactin, subtilosin A) and quorum quenching.	Increased survival of shrimp against pathogenic *Vibrio* spp.	[122]
*Bacillus licheniformis & B. subtilis* (Mix probiotic)	3.5 × 10⁸ CFU/g.m^−3^ (in water)	Post-larvae	Bioremediation (NH₃ degradation) and competitive exclusion, leading to improved water quality and reduced *Vibrio* counts.	Significantly lower *Vibrio* counts in water and improved shrimp survival compared to control.	[123]
*Bacillus subtilis & B. cereus* (mix probiotic)	7×10⁶ & 2.5×10⁷ CFU.g⁻¹ feed	Juvenile	Competitive exclusion, enhanced THC and clearance efficiency	86.7% survival vs. 26.7% control	[124]
*Bacillus licheniformis* (ATCC 11946)	1×10^8^ CFU.g⁻¹ feed	Juvenile	Serum and hepatic immune and antioxidant enzyme activities, intestinal morphology, assembly of intestinal microbiota by yielding more beneficial bacteria and reducing the abundance of *Virbio*	67% survival in *V. parahaemolyticus* challenge vs. 32% in control	[64]
*Bacillus licheniformis* (BCR 4-3)	1 ×10^6^ CFU L^−1^ (Culture water) + 1 ×10^6^ CFU.g^−1^ Feed	Juvenile	Increased growth performance and the mRNA of lysozyme, crustin, penaeidin4 and superoxide dismutase (SOD) genes significantly upregulated	87.5% survival in *V. parahaemolyticus* challenge vs. 58.3% in control	[125]
*Bacillus velezensis* (BV007)	1 × 10⁷ CFU.g⁻¹ feed	Juvenile	Enhanced immune response (respiratory burst, SOD, CAT, AKP), increased abundance of beneficial bacteria (*Bacillus*), and reduced pathogenic *Vibrio* in intestine.	30.6% cumulative mortality after V. parahaemolyticus challenge vs. 97.2% in control.	[126]
*Bacillus velezensis* (DH82)	1 × 10⁵ CFU.mL⁻¹ (in water)	Juvenile	Quorum quenching (AHL degradation) and production of antimicrobial peptides. Reduced expression of *V. parahaemolyticus* virulence genes (*luxR, toxR, vhhA, flaB*).	79.9% survival post-*V. parahaemolyticus* challenge vs. 39.5% in control.	[17]
*Bacillus pumilus* (Sonora)	∼2.5 × 10⁸ CFU·g⁻¹ feed	Juvenile	Produces antimicrobial peptides (surfactin, fengycin, bacilysin)	11.1% mortality vs. 90% control	[127]
*Bacillus coagulans* (ATCC 7050)	1 × 10⁸ CFU.g⁻¹ feed	Juvenile	Increased digestive enzyme activity (trypsin, lipase, amylase), improved intestinal morphology (villus height), and enhanced immune response.	76% survival post-*V. parahaemolyticus* challenge vs. 40% in control.	[106]
*Bacillus amyloliquefaciens* (TOA5001)	5 × 10⁶ CFU.g⁻¹ feed	Juvenile	Probiotic feeding altered microbiota and increased bacterial diversity, potentially inhibiting *Vibrio* through competition.	Significantly higher survival in probiotic-fed group after *V. parahaemolyticus* (AHPND) immersion challenge.	[128]
*Lactobacillus plantarum* (CMT1)	1 × 10⁸ CFU.kg⁻¹ diet	Juvenile	Competitive exclusion, bacteriocins, organic acids, immune enhancement	83.3% survival vs. 71.7% control	[129]
*Lactobacillus plantarum* (Ep-M17)	5 × 10⁸ CFU.g⁻¹ feed	Juvenile	Increased digestive and immune enzymes (trypsin, SOD, CAT, TRY, AKP, LIP, and AMS), and activate significantly differential expression of genes in immune, nutritional, metabolic, and Signal Transduction-related pathways in the gut of shrimp	76.9% protection rate against *V. parahaemolyticus*.	[107]
*Pseudoalteromonas* spp**.** (CDM8, CDA22)	1 × 10⁷ CFU.kg⁻¹ feed	Juvenile	Competitive exclusion, production of antimicrobial compounds, and enhancement of the host's immune system.	96.7% mortality of *V. parahaemolyticus*-challenged control vs. significantly lower mortality in probiotic-fed group.	[130]
*Clostridium butyricum* (CBG01)	1 × 10¹¹ CFU·kg⁻¹ feed	Juvenile	Alkaline phosphatase, acid phosphatase, total nitric oxide synthase, lysozyme, peroxidase, superoxide dismutase activities, total antioxidant capacity, and phenonoloxidase content in the serum and the relative expression levels of SOD, LZM, proPO, LGBP, HSP70, Imd, Toll, Relish, TOR, 4E-BP, eIF4E1α, eIF4E2 genes in the hepatopancreas significantly enhanced	50% reduction in mortality	[131]
*Lactococcus lactis* (FA1)	1 × 10⁸ CFU.ml⁻¹ (1 ml bacteria sprayed over 2 g feed)	Post-larvae	Competitive exclusion, production of bacteriocins, and immunostimulation.	65% survival in probiotic-treated infected group vs. 45% in untreated infected group.	[132]
*Pediococcus pentosaceus* (R6)	1 × 10⁷ CFU.g⁻¹ diet (with Fructooligosaccharide)	Juvenile	Enhanced immune parameters (THC, PO, lysozyme, phagocytosis)	80% survival vs. 20% control	[19]
*Halomonas alkaliphila* (synbiotic)	1 × 10⁹ CFU.kg⁻¹ feed	Post-larvae	Quorum quenching (lactonase), prebiotic enhancement	79.9% survival vs. 39.5% control	[90]
*Exiguobacterium acetylicum*	1.58 × 10⁷ CFU.ml⁻¹ (in water)	Juvenile	Quorum quenching (aiiA gene), enzymatic activity	90.9% survival vs. 70.9% control	[133]
*Paenibacillus polymyxa* (ATCC 842)	1 × 10⁸ CFU.g⁻¹ feed	Juvenile	Enhanced immune gene expression, reduced opportunistic pathogens	78.3% survival vs. 20% control	[88]
*Cobetia sp.* (W1B)	1 × 10⁶ CFU.ml⁻¹ (in water)	Juvenile	Quorum quenching, down-regulation of V. harveyi virulence genes	47.4% reduction in cumulative mortality	[95]
*Arthrobacter bussei*	10^7^ CFU.kg^−1^ with encapsulated and 10^8^ CFU.kg^−1^ without encapsulated (0.2-1.0% in feed)	Juvenile	Enhanced antioxidant capacity (SOD, CAT), innate immunity, and growth performance.	Increased immune parameters and disease resistance against *V. alginolyticus* and *V. parahaemolyticus*.	[108]

Additionally, probiotics play a critical role in modulating the immune system of shrimp. As invertebrates, shrimp rely primarily on innate immune defenses to combat infections [134]. Probiotic supplementation has been shown to enhance several immune parameters, including phenoloxidase activity, antimicrobial peptide production, and antioxidant enzyme activity. These physiological improvements increase the ability of shrimp to resist pathogenic infections [135].

### Competitive exclusion and microbial niche occupation

**Fig. 2:**
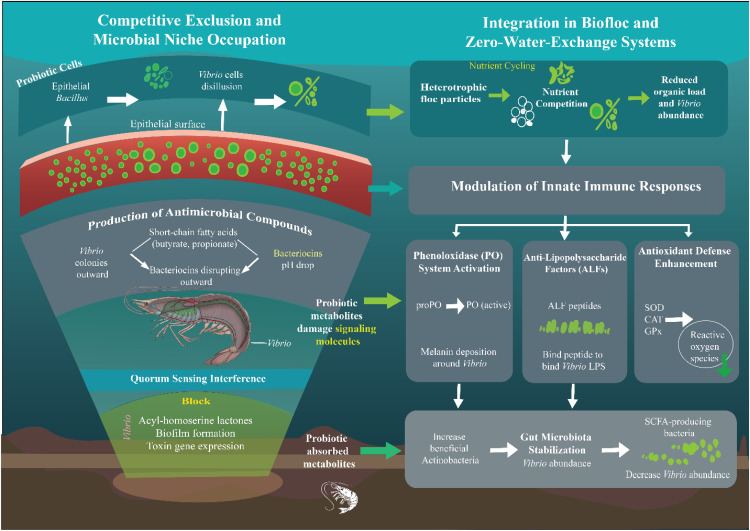
Probiotic suppression of *Vibrio* in *Litopenaeus vannamei* involves competitive exclusion, secretion of antimicrobial compounds (bacteriocins/SCFAs), and quorum quenching, alongside organic-load reduction in biofloc and zero-water exchange systems. Probiotics further enhance immunity via activation of proPO, stimulation of ALFs, and upregulation of antioxidant enzymes (SOD, CAT, GPx), while stabilizing gut microbiota by promoting beneficial bacteria and suppressing *Vibrio*. Here, SOD– superoxide dismutase; CAT– catalase; GPx– glutathione peroxidase; LPS– lipopolysaccharide; SCFA– short-chain fatty acid; ALF– anti-lipopolysaccharide factor.

Competitive exclusion represents one of the most important mechanisms through which probiotics inhibit pathogenic microorganisms in aquaculture systems. This concept is based on the principle that beneficial microbes can occupy ecological niches within the host or environment, thereby preventing the colonization and proliferation of pathogenic bacteria such as *Vibrio* species [136,137] (Fig. 2). In shrimp culture systems, the gastrointestinal tract and surrounding aquatic environment contain complex microbial communities that compete for limited nutrients and space [114,138]. When probiotic microorganisms are introduced into the system, they can rapidly colonize these niches and establish stable microbial populations. This colonization limits the ability of pathogenic bacteria to attach to host tissues or proliferate within the digestive tract [114,115,[139], [140]]. Certain probiotic strains are particularly effective at adhering to the intestinal mucosa of shrimp, forming protective microbial layers that block pathogen attachment sites. This physical barrier can significantly reduce the likelihood of infection by opportunistic pathogens such as *Vibrio harveyi* and *Vibrio parahaemolyticus* [139,141,142].

In addition to occupying attachment sites, probiotics compete with pathogens for essential nutrients required for growth and reproduction [143,144]. By efficiently utilizing available nutrients, beneficial microorganisms reduce the resources available for pathogenic bacteria, thereby suppressing their proliferation [[122], [145]]. Competitive exclusion can also occur at the environmental level within shrimp culture ponds. When probiotic bacteria are applied directly to the water, they can alter the microbial composition of the culture environment, promoting the dominance of beneficial microbial communities [146]. This microbial balance helps prevent the rapid expansion of pathogenic bacteria and contributes to a healthier culture ecosystem [147]. The effectiveness of competitive exclusion depends on several factors, including the probiotic strain used, environmental conditions, and the stability of the microbial community [76]. Nevertheless, this mechanism plays a fundamental role in the ability of probiotics to control *Vibrio* populations and reduce disease risk in shrimp aquaculture systems [71].

### Production of antimicrobial compounds

Another important mechanism by which probiotics inhibit pathogenic bacteria is the production of antimicrobial compounds. Many probiotic microorganisms synthesize bioactive substances that can directly suppress the growth or activity of pathogenic microbes, including various *Vibrio* species [148,44] (Fig. 2). Among the most commonly produced antimicrobial substances are bacteriocins, which are small peptide molecules capable of inhibiting closely related bacterial species. Bacteriocins produced by probiotic bacteria can disrupt bacterial cell membranes, inhibit essential metabolic processes, and ultimately lead to the death of pathogenic microorganisms [149]. Notably, nisin (produced by lactic acid bacteria) and subtilosin A, produced by *Bacillus subtilis*, is a cyclic bacteriocin that exerts antimicrobial activity by interacting with bacterial membranes and causing membrane permeabilization, thereby disrupting membrane integrity and cellular function [150,151]. Similarly, lacticin and lactococcin, synthesized by lactic acid bacteria, create pores in the cell membrane that inhibit or eliminate their target pathogenic microorganisms [150]. Furthermore, Pediocin, produced by *Pediococcus* spp., primarily inhibits Gram-positive bacteria by binding to susceptible cells and dissipating the membrane potential and proton motive force. Activity against Gram-negative pathogens is generally limited unless the outer membrane is first destabilized by a synergistic factor, which allows pediocin to access the cytoplasmic membrane [152]. Conversely, *Bacillus* strains produce cyclic lipopeptides, including surfactin, iturin, and fengycin, which exert antimicrobial activity mainly by disrupting cell membranes, leading to membrane leakage and lysis of susceptible pathogens [153]. In addition to bacteriocins, probiotics may produce organic acids such as lactic acid, acetic acid, and butyric acid [[154], [155], [156], [157]]. These organic acids can reduce the pH of the surrounding environment, creating conditions that are unfavorable for the growth of many pathogenic bacteria. Lower pH levels can disrupt cellular processes within pathogens and reduce their virulence [158]. Some probiotic bacteria also generate hydrogen peroxide and other reactive compounds with antimicrobial properties. These substances can damage bacterial cell membranes, proteins, and nucleic acids, thereby inhibiting the proliferation of pathogenic microbes [159]. Enzymatic activity represents another mechanism of antimicrobial action. Certain probiotic microorganisms produce extracellular enzymes capable of degrading bacterial cell walls or interfering with pathogen metabolism. These enzymatic processes contribute to the suppression of pathogenic bacterial populations within aquaculture systems. The combined production of multiple antimicrobial compounds by probiotic bacteria provides a powerful biological defense against pathogenic microorganisms. This multifaceted antimicrobial activity plays a critical role in reducing the abundance of *Vibrio* species in shrimp culture environments and protecting shrimp from bacterial infections.

### Quorum sensing interference

Quorum sensing (QS) is a bacterial communication system that enables microorganisms to coordinate collective behaviors through the production and detection of signaling molecules known as autoinducers [160,161]. At the molecular level, QS in many pathogenic bacteria including *Vibrio* species, begins with the synthesis of autoinducers (AHLs via LuxI/SAM, processed AIPs, or AI-2), which accumulate extracellularly until they bind cognate receptors (LuxR-type regulators or two-component sensors like AgrC) to initiate transcription of virulence factors, biofilm components, and secretion systems [[162], [163], [164], [165], [166], [167], [168], [169], [170]]. In shrimp aquaculture systems, QS also plays a crucial role in the pathogenicity of *Vibrio* bacteria [171]. When bacterial populations reach a critical density, the concentration of signaling molecules increases, triggering the coordinated activation of genes associated with virulence and infection processes [[172], [173]] (Fig. 2).

Probiotics can interfere with this communication system through a process known as quorum quenching [174]. Quorum quenching (QQ) disables communication by targeting specific enzymatic and receptor steps [175,176]. Enzymatic degradation via lactonases opens the homoserine-lactone ring, acylases/amidases cleave the amide bond to remove the acyl tail, and oxidoreductases modify oxo groups, converting active signals into inert forms [[175], [176], [177], [178]]. Chemical QS inhibitors may block synthase activity (e.g., SAM analogs or MTAN/LuxS inhibitors) or serve as receptor antagonists (e.g., halogenated furanones) that prevent proper dimerization and DNA binding [179,180]. Additional molecular strategies include blocking signal transport by extracellularly phosphorylating AI-2 via the LsrK enzyme, or inhibiting processing enzymes like AgrB, alongside host enzymes like paraoxonases that hydrolyze AHLs at mucosal surfaces [174]. As a result, receptor activation is reduced, downregulating QS regulons and impairing biofilm formation, toxin production, and horizontal gene transfer, making the pathogen more vulnerable to antibiotics and host immunity [[174], [181]]. QS interference therefore represents an important mechanism through which probiotics contribute to disease prevention in aquaculture. By disrupting bacterial communication systems, probiotics can suppress virulence expression and reduce the ability of pathogenic bacteria to cause infections in cultured aquatic populations [[158], [182]]. Nevertheless, because QS systems differ markedly by species, QQ agents must be precisely matched; moreover, pathogens may evolve resistance through efflux pumps, QS-independent regulatory pathways, while disrupting interspecies (AI-2) signaling could unintentionally disturb beneficial microbial communities [[174], [183]].

### Modulation of innate immune responses

Shrimp possess an innate immune system that serves as their primary defense against pathogenic microorganisms. Unlike vertebrates, shrimp lack an adaptive immune system and therefore rely heavily on innate immune responses (epigenetic, mRNA and microRNA-based) to recognize and eliminate pathogens [[184], [185], [186], [187]]. Probiotics have been shown to enhance several components of this immune system, thereby improving disease resistance in cultured shrimp (Fig. 2). One of the key immune responses stimulated by probiotics is the activation of hemocytes, which are immune cells responsible for pathogen recognition and elimination [[3], [188]]. Probiotic supplementation can increase hemocyte counts and enhance their phagocytic activity, enabling shrimp to more effectively capture and destroy invading microorganisms [189].

Probiotics also stimulate the production of antimicrobial peptides and other immune-related molecules [190]. Mostly, penaeidins, whey acidic protein (WAP) domain containing proteins [crustins and single WAP domain-containing peptides (SWD)], anti-lipopolysaccharide factors (ALFs), histones, hemocyanin, lysozymes, a C-type lectin, and peritrophins are commonly observed in shrimp [[191], [192], [193], [194], [195], [196], [197]]. These compounds play important roles in neutralizing pathogenic bacteria and preventing their proliferation within the host organism [198].

In addition, probiotics can enhance the activity of enzymes involved in immune defense mechanisms. Increased enzymatic activity improves the shrimp’s ability to respond rapidly to infections and limit pathogen spread within tissues [190,[199], [200], [201]]. Another important effect of probiotics is the reduction of physiological stress in shrimp. Stress can weaken immune responses and increase susceptibility to disease [202]. By improving gut health, nutrient absorption, and microbial balance, probiotics contribute to overall physiological stability and stronger immune function [203]. Through these mechanisms, probiotic supplementation strengthens the innate immune system of shrimp and enhances their capacity to resist infections caused by pathogenic *Vibrio* species [125].

#### Phenoloxidase (PO) system activation

The phenoloxidase (PO) system is a crucial component of the innate immune defense mechanism in shrimp. This enzymatic pathway plays a key role in pathogen recognition, melanization, and encapsulation processes that help eliminate invading microorganisms [204]. Activation of the PO system leads to the production of melanin, which forms protective barriers around pathogens and contributes to their neutralization [205]. Several studies have demonstrated that probiotic supplementation can significantly enhance phenoloxidase activity in *L. vannamei* [206]. Increased PO activity improves the shrimp’s ability to respond rapidly to microbial infections and prevents the proliferation of pathogenic bacteria within tissues [187]. Probiotic microorganisms may stimulate the PO system through interactions with immune receptors present on hemocytes [134]. These interactions trigger signaling pathways that activate the prophenoloxidase cascade, leading to increased enzyme activity and enhanced immune defense (Fig. 2). Enhanced PO activity has been associated with improved disease resistance in shrimp exposed to pathogenic bacteria such as *Vibrio harveyi* [207]. By strengthening this critical immune pathway, probiotics contribute to more effective protection against bacterial infections in aquaculture systems.

#### Anti-lipopolysaccharide factors (ALFs)

Anti-lipopolysaccharide factors (ALFs) are antimicrobial peptides that play an essential role in the immune defense of crustaceans [208]. These molecules possess strong antibacterial activity against Gram-negative bacteria, including many pathogenic *Vibrio* species commonly found in shrimp aquaculture systems [209,210]. ALFs function by binding to lipopolysaccharides present in the outer membrane of Gram-negative bacteria. This interaction disrupts bacterial cell membrane integrity and inhibits microbial growth [211]. As a result, ALFs serve as an important component of the shrimp immune response against bacterial infections [212]. Probiotic supplementation has been shown to enhance the expression of genes responsible for the production of ALFs in shrimp (Fig. 2). Increased ALF production strengthens the shrimp’s antimicrobial defense and reduces the ability of pathogenic bacteria to establish infections within the host organism. The stimulation of antimicrobial peptide production represents an important immunomodulatory effect of probiotics in aquaculture systems. By enhancing the expression of ALFs and other immune molecules, probiotics contribute to improved resistance against *Vibrio* related diseases in cultured shrimp [48].

#### Antioxidant defense enhancement

Oxidative stress is a common physiological challenge faced by shrimp cultured under intensive aquaculture conditions. Environmental stressors such as poor water quality, high stocking densities, and pathogen exposure can lead to the excessive production of reactive oxygen species (ROS) within shrimp tissues [213]. Excessive ROS accumulation can damage cellular components including proteins, lipids, and DNA, ultimately impairing immune function and increasing susceptibility to disease [[200], [214], [215], [216]]. To counteract these harmful effects, shrimp possess antioxidant defense systems composed of enzymes such as superoxide dismutase (SOD), catalase (CAT), and glutathione peroxidase (GPx) [[217], [218], [219], [220]] (Fig. 2). Probiotic microorganisms have been shown to enhance the activity of these antioxidant enzymes in shrimp. Increased antioxidant capacity helps neutralize reactive oxygen species and reduces cellular damage caused by oxidative stress [221]. By strengthening antioxidant defenses, probiotics improve the physiological resilience of shrimp and support overall health under intensive farming conditions. This protective effect contributes to improved growth performance, reduced stress responses, and enhanced resistance to bacterial infections [92].

### Gut microbiota stabilization

The gastrointestinal tract of shrimp hosts a complex and dynamic microbial community that plays a vital role in digestion, nutrient absorption, immune regulation, and disease resistance. Maintaining a balanced gut microbiota is essential for optimal shrimp health and growth performance [[49], [50], [51]]. Disruptions in the microbial composition of the shrimp gut can create opportunities for opportunistic pathogens such as *Vibrio* species to proliferate [222,49,223]. Factors including environmental stress, poor water quality, and inappropriate feed formulations may contribute to microbial imbalances within the digestive tract [224]. Probiotic supplementation can help restore and maintain microbial equilibrium within the shrimp gut [225,226] (Fig. 2). Beneficial microorganisms introduced through probiotics can colonize the gastrointestinal tract and promote the dominance of beneficial bacterial populations. This microbial balance reduces the likelihood of pathogen colonization and supports healthy digestive processes [114,138]. In addition to suppressing pathogenic bacteria, probiotics can enhance digestive efficiency by producing enzymes that aid in the breakdown of complex nutrients [118]. Improved nutrient digestion leads to better feed utilization and increased growth performance in cultured shrimp. Probiotics may also stimulate the production of beneficial metabolites such as short-chain fatty acids that support intestinal health and immune function [48]. These metabolites contribute to the maintenance of intestinal integrity and enhance the shrimp’s ability to resist infections. Overall, the stabilization of gut microbiota represents a key mechanism through which probiotics improve shrimp health and reduce disease susceptibility in aquaculture systems [120].

### Integration in biofloc and zero-water-exchange systems

Biofloc technology and zero-water-exchange culture systems have emerged as innovative approaches for sustainable shrimp aquaculture. These systems rely heavily on microbial processes to maintain water quality and recycle nutrients within the culture environment [227,228]. In biofloc systems, microbial aggregates composed of bacteria, algae, protozoa, and organic particles form suspended flocs within the water column. These flocs serve as both biological filters and supplementary feed sources for shrimp. Beneficial bacteria within the biofloc community can convert nitrogenous waste products such as ammonia into microbial biomass, thereby improving water quality [229,230]. Probiotics can be effectively integrated into biofloc systems to enhance microbial stability and suppress pathogenic bacteria [231]. The introduction of beneficial microorganisms helps maintain a balanced microbial community and prevents the dominance of harmful bacteria such as pathogenic *Vibrio* species [227] (Fig. 2).

Zero-water-exchange systems also benefit from probiotic supplementation. In these systems, water exchange is minimized to reduce environmental impacts and prevent the introduction of external pathogens [232,233]. However, maintaining microbial balance becomes particularly important in such closed systems. Probiotics can contribute to improved nutrient cycling, organic matter degradation, and pathogen suppression within these systems. By supporting stable microbial ecosystems, probiotics help maintain favorable environmental conditions and promote sustainable shrimp production. The integration of probiotics with advanced aquaculture technologies therefore represents a promising strategy for improving productivity, enhancing disease resistance, and reducing environmental impacts in *L. vannamei* farming [224].

## Conclusions

The Pacific white shrimp *L. vannamei* plays a central role in global aquaculture production. However, the rapid intensification of shrimp farming has increased the prevalence of infectious diseases, particularly those caused by pathogenic *Vibrio* species. Traditional reliance on antibiotics for disease control has raised significant concerns regarding antimicrobial resistance, environmental contamination, and food safety. Consequently, alternative disease management strategies have become increasingly important for sustainable shrimp aquaculture. Probiotics represent one of the most promising biological tools for controlling bacterial pathogens in shrimp culture systems. Through mechanisms such as competitive exclusion, antimicrobial compound production, quorum sensing interference, immune modulation, and gut microbiota stabilization, probiotics can effectively suppress pathogenic *Vibrio* populations and enhance shrimp health. The integration of probiotics with modern aquaculture technologies such as biofloc systems and recirculating aquaculture systems further enhances their potential for sustainable disease management. Continued research is required to optimize probiotic strains, application methods, and environmental compatibility. With appropriate implementation, probiotic-based strategies may significantly contribute to the sustainable development and long-term productivity of shrimp aquaculture worldwide.

## Declaration of generative AI and AI-assisted technologies in the manuscript preparation process

During the preparation of this work the authors used ChatGPT in order to refine the language of the manuscript. After using this tool/service, the authors reviewed and edited the content as needed and take full responsibility for the content of the published article.

## CRediT authorship contribution statement

**Tashrif Mahmud Minhaz:** Writing – original draft, Supervision, Methodology, Investigation, Conceptualization. **Sayeed Hasan Al Banna Tanim:** Writing – original draft, Investigation. **Samiya Sultana Lima:** Writing – original draft, Investigation. **Shahadat Hossain:** Writing – review & editing, Resources, Methodology. **Zahidul Islam:** Writing – review & editing, Methodology, Investigation. **Sadia Afrin:** Writing – review & editing, Investigation. **Md Azharul Hoque Shakil:** Writing – review & editing, Investigation.

## Declaration of competing interest

Authors have no competing interest to declare for this work.

## Data Availability

No data was used for the research described in the article.
